# Supplementary material for: A set of multi-entry identification keys to African frugivorous flies (Diptera, Tephritidae)
Source: Zookeys. 2014 Jul 24;(428):97–108. doi: 10.3897/zookeys.428.7366 (PMC4143993; doi:10.3897/zookeys.428.7366)
Supplement: Supplementary material 6 — Key to Ceratitis [file zookeys-428-097-s006.zip › SF6_ZooKeys_key to Ceratitis/key/SF6_ZooKeys_key to Ceratitis/Media/Html/Ceratitis lentigera .htm]

Ceratitis (Ceratalaspis) lentigera Munro


***Ceratitis*** **(*Ceratalaspis*)
*lentigera* Munro**

*Ceratitis* (*Pardalaspis*) *lentigera* Munro, 1933b: 8.

Body
length: 4.51 (3.85-5.40) mm; wing length: 4.28 (3.75-4.85) mm.

Male

Head.
Antenna yellow-orange. Third antennal segment three times as long as second
segment. Arista with short to medium length hairs over entire length. Antennal
inplant with dark horizontal band. Frons yellow with dark median band; with
short scattered hairs which are largely the same colour as frons; convex but
not distinct, in lateral view not distinctly projecting forwards at antennal
implant. Face yellow-white with dark median band; lower eye-margin with
slightly darker colouration, not distinct patch. Chaetotaxy normal for
subgenus. Bands on face and frons not always well pronounced.

Thorax.
Postpronotum white, spotted. Ground colour of mesonotum pale with orange tinge;
mesonotum, colouration along median line variable. Chaetotaxy normal for
subgenus. Outer scapular setae black, inner ones pale (sometimes all black).
One anepisternal bristle. Scutellum white basally, otherwise yellow with three
black separate markings apically; with two separate dark spots placed
subbassaly, hardly touching base of scutellum. Subscutellum with three black
spots at same position as scutellar spots, dorsally narrowly joining.

Legs
yellow; setation typical for subgenus, mainly pale especially on femora.
Posterior and posterodorsal rows on fore femur pale.

Wing
bands with extensively yellow markings, sometimes more yellow-brown. Banding,
marginal band continuous; cubital band free, medial band absent; crossvein r-m
just before middle of discal cell. Crossvein dm-cu posteriorly more inwards
than anteriorly.

Abdomen.
Pale yellow. with few spots, no distinct banding.

Female

As
male except for the following characters: Facial and frontal bands sometimes
less clear, and interrupted. Mesonotum, orange tinge sometimes very faint.
Abdomen greyish brown. Oviscape shorter than abdominal terga 3-6 combined.

(Description
after De Meyer, 1998)
